# Predictors of Adherence to Treatment in Behavioral Health Therapy for Latino Immigrants: The Importance of Trust

**DOI:** 10.3389/fpsyt.2019.00817

**Published:** 2019-11-08

**Authors:** Irene Falgas-Bague, Ye Wang, Souvik Banerjee, Naomi Ali, Karissa DiMarzio, Diego Palao Vidal, Margarita Alegría

**Affiliations:** ^1^Disparities Research Unit, Department of Medicine, Massachusetts General Hospital, Boston, MA, United States; ^2^Department of Psychiatry and Forensic Medicine, Universitat Autònoma de Barcelona, Bellaterra, Spain; ^3^Department of Mental Health, University Hospital Parc Tauli-I3PT, Sabadell, Spain; ^4^CIBERSAM, Barcelona, Spain; ^5^Departments of Medicine and Psychiatry, Harvard Medical School, Boston, MA, United States

**Keywords:** Latinos, co-occurring disorders, barriers, adherence to treatment, ethnicity, immigrant

## Abstract

A complex array of barriers to care influence patients' adherence to behavioral healthcare services. Understanding barriers to care is critical to ensure sufficient dosage of treatment. This study assessed the influence of perceived barriers on Latino migrants' prospective adherence to treatment for co-occurring mental health and substance use disorders as part of a clinical trial. Eligible participants (18–70 years-old) were recruited from community-based settings and classified according to their intervention session attendance. Baseline assessments included socio-demographic factors, clinical characteristics (i.e., depression, anxiety, post-traumatic stress disorder, substance use), psychosocial and cultural factors (i.e., ethnic identity, health literacy, discrimination), and perceived attitudinal and structural barriers to care. Treatment involved 10-sessions of cognitive-behavioral therapy, psychoeducation, and mindfulness (Integrated Intervention for Dual problems and Early Action) and emphasized participant's engagement in treatment. We used multinomial logistic regression models to examine the association between barriers to care reported at baseline, sociodemographic characteristics, psychosocial and cultural factors, clinical factors, and treatment adherence. Mistrust in previous behavioral health treatment(s) was the reported barrier significantly associated with completion of the program after adjusting for clinical, psychosocial, and cultural factors, with those expressing mistrust in previous treatment(s) showing higher rates of completion compared to those who did not report this barrier. Evidence-based and culturally-tailored interventions provided by ethnically matched providers may overcome cultural mistrust and increase adherence to behavioral health care among Latino immigrants.

## Introduction

Latinos represent the largest immigrant population in the United States (U.S.) and Spain (1). The growth of first- and second-generation Latinos in both countries poses challenges for public health systems, from limited accessibility to behavioral health services (2, 3) to low quality and continuity of care (4). Although disparities in behavioral health care among Latinos have been well-documented in the U.S. (5), there is a paucity of research in both countries identifying factors that might shape adherence to behavioral health treatments among Latino populations. A complex array of psychological, cultural and sociodemographic factors influences an individual's entry and adherence to behavioral health care (6). Thus, identifying salient barriers to care is a critical task for clinicians and administrators.

Existing literature suggests that individuals with co-occurring mental health and substance use disorders (COD) access behavioral health treatment at extremely low rates compared to individuals without co-morbidities (7). For example, only 9.1% of these individuals receive treatment for both disorders, while 52.5% receive no treatment at all (8). Yet, integrated treatments are among the most effective interventions for persons with COD (9, 10). Nevertheless, Latinos face unique barriers inhibiting access to specialized treatment (11). Research has shown that Latinos have lower rates of treatment adherence, attending fewer sessions and prematurely dropping out of cognitive behavioral therapy (CBT) compared to non-Latino Whites (12). These differences have been attributed to the double stigma associated with mental illness and being an ethnic minority, logistical, motivational, and attitudinal factors (13–15). For example, a previous study found that approximately 50% of Latino immigrants reported self-reliant attitudes regarding behavioral health care (e.g., wanting to handle problems on one's own) and structural barriers (e.g., difficulties with transportation and scheduling flexibility). The study identified these barriers as significant obstacles to treatment adherence (16). Even if Latino immigrants view their symptoms as warranting attention, they are unlikely to seek treatment if they do not believe they will benefit from professional services.

According to Andersen-Newman's healthcare utilization model (17), predisposing (e.g., sociodemographic characteristics and attitudes toward health care), enabling (e.g. employment), and clinical factors (e.g. symptoms and severity) determine an individual's ability to cope and command resources to deal with problems, shaping his/her use of services (18). An extension of this model includes psychosocial factors such as discrimination and health literacy to better comprehend the complex role of race/ethnicity in help-seeking (19, 20). Yet, the literature is inconclusive with regards to how these factors predict adherence to treatment.

One strategy that has been effective in improving adherence among Latino populations is culturally adapting interventions (21). To this end, we conducted a multisite randomized trial of the “Integrated Intervention for Dual Problems and Early Action” (22), which was specifically designed to treat immigrant and U.S./Spain-born Latinos with COD (NIDA Grant R01DA034952). The trial was implemented at three sites (Boston, Madrid, and Barcelona) and incorporated a patient-centered, community-based approach. Treatment took place in-person or by phone and included extended outreach on Saturdays and Sundays.

The current study aimed to assess predictors of treatment adherence in a large sample of Latino immigrants with COD. There are several aspects that make our study novel. First, we assess several barriers before immigrants enter treatment to determine if they predict individuals' adherence to care. Second, we include immigrants from two countries, which allows us to better assess characteristics that encourage adherence in immigrant populations. Third, we include a wide array of factors to determine if barriers remain important factors after adjusting for sociodemographic, clinical, and psychosocial characteristics in a heterogeneous sample of Latino immigrants. To do this, we evaluated predictors of adherence to treatment based on the three groups that best represented the distribution of patient participation into the Integrated Intervention for Dual problems and Early Action (IIDEA) intervention: 0–1 sessions (tercile 1 or reference category), 2–9 sessions (tercile 2), and 10–12 sessions (tercile 3) (see **Figure 1s** in **Supplementary Material**).

## Methods

### Study Design and Setting

Data were drawn from baseline interviews and clinical trial data collected for the International Latino Research Partnership, a multisite study that sought to improve behavioral health care services for Latino migrants (22). Participants were enrolled between September 2014 and February 2017 through direct contact in clinic waiting rooms, emergency departments, community-based organizations, or through healthcare professionals and participant referrals. Participating clinics were affiliated with large safety-net health care systems serving diverse populations. All participants provided informed consent before participating in the trial. The study was approved by the review boards of all participating institutions.

### Participants

Eligible participants were those between 18–70 years old who self-identified as Latino (from any Spanish-speaking country—Caribbean, Central America and Mexico, or South America), and who had migrated to the U.S. or Spain. Participants had to screen positive for comorbid mental health and substance misuse (COD) but report not currently receiving specialty behavioral health services (i.e., therapy with a psychiatrist, psychologist, or social worker) in the past 3 months or upcoming month. Positive COD screening included an affirmative response to two questions about mental health and two about substance use on the AC-OK screener (23), a 15-item questionnaire validated in Spanish (24). Sensitivity and specificity of the questionnaire was shown to be consistent with standardized screeners (24). Internal consistency was comparable to the original English sample (α = 0.82 for mental health and α = 0.90 for substance abuse). Participants were excluded only if they lacked capacity to consent, assessed by a validated screener (25), or reported suicidal ideation, assessed with the Paykel Suicide Scale (26).

### Procedures

For the study, 2,284 prospective participants were approached directly by the research team in clinic waiting rooms or were referred by primary care providers or staff from community agencies and then contacted by phone. Of these, 384 were eligible to participate and 341 agreed to a baseline interview. Trained interviewers conducted research assessments in Spanish or English depending on participants' preferred language. Interviews were audio-recorded and lasted approximately 1 hour. Participants were compensated with $40/€30 gift cards for the assessment, but not for participating in treatment sessions. Upon completion of the baseline assessment, participants were randomized to the IIDEA intervention or enhanced usual care as part of the control group. Participants in the control group were contacted four times throughout the intervention period by a care manager, who administered the brief assessment used in clinical sessions and monitored each patient's mental health to assess increases in symptoms that might require specialized care. A member of the research team contacted those randomized to the intervention group (n = 172) to schedule the first appointment with a clinician. Clinicians were also randomized per patients' available times for treatment.

### Intervention

IIDEA was designed to maintain fidelity to evidence-based approaches while addressing a range of mental health conditions (e.g., depression, anxiety, traumatic stress, mild to moderate drug/alcohol abuse) in Latino adults. IIDEA is a brief, transdiagnostic therapy model adapted to engage and retain Latino participants in treatment. The program involves 10 weekly sessions delivered over 3–4 months by trained clinicians who attend weekly supervision meetings. Depending on participant's needs, two extra sessions were offered at the end of the program. IIDEA integrates elements of CBT, motivational interviewing, relapse prevention, assertive communication, and HIV/STD risk prevention. The program also includes basic elements of the cultural formulation model to improve participant engagement in treatment. Clinicians included psychiatric residents, clinical psychologists, and social workers. All clinicians were fluent Spanish speakers, with five of the 14 total clinicians self-identifying as Latinos; the remaining nine clinicians were Spanish natives. Depending on the participant's preferences, the intervention was delivered in-person at a clinic or community setting or over the phone. The goals of the program were to: engage, elicit, and improve coping skills to reduce symptoms of depression, anxiety, and PTSD; improve assertive communication skills; reduce or eliminate alcohol and substance use problems; and reduce HIV/STD risk behaviors.

### Measurements

#### Adherence to IIDEA Intervention

We measured adherence by the number of sessions attended. We first explored the distribution of participants according to attendance to inform how to group the participants (see **Figure 1s** in **Supplementary Material**). The first group (tercile 1) comprised participants who either never started the intervention or attended only one session. The second group (tercile 2) included those who had completed two to nine sessions, and the third group (tercile 3) included those who completed 10 or more sessions.

#### Sociodemographic and Predisposing Factors

Demographic factors included age, gender, and site of recruitment (Barcelona, Madrid, or Boston). We also collected self-reported race/ethnicity (White, Black, Indigenous/Native American, Hispanic/Latino/Caribbean, and Mixed Race), highest education level obtained, socioeconomic status (classified into two categories; total personal annual income >$15,000 or <$15,000), immigration status (defined by citizen or non-citizen to host country) and employment status (1 if employed, including part time; 0, otherwise).

#### Psychosocial and Cultural Factors

We assessed individuals' perceived discrimination, intercultural family conflict, health literacy, time in the host country, and number of visits to their country of origin as psychosocial and cultural factors that could predict adherence to the intervention. Participants completed the Everyday Discrimination Scale (EDS; range = 9 to 54) (27, 28), previously used in the National Latino and Asian American Study (α = 0.82) (29). The EDS assessed how often participants experienced unfair treatment in their day-to-day life. Response categories ranged from 1 (never) to 6 (almost every day), with higher scores indicating greater perceived discrimination. To assess intercultural family conflict, we used four items of the Family/Culture Stress subscale of the Hispanic Stress Inventory (range = 0 to 10) (α = 0.67) (30), designed to measure family interference with personal goals, arguments with family members, and the breakdown of the family unit. Sense of belonging was also assessed using one item (coded as yes = 1, otherwise = 0) from the Family/Culture Stress subscale (30). The 3-item Ethnic Identity Scale (α = 0.73), derived from the 35-item Cultural Identity Scale for Latino Adolescents (range = 3–12) (31), was used to gauge cultural identity, with questions pertaining to participants' self-identified culture or racial/ethnic group. We also collected data on the amount of time participants had spent in their host country (US or Spain) and number of visits to their country of origin in the past 12 months. We assessed health literacy with three questions from the Health Literacy Screening Questionnaire (range = 3–15) (32), which assessed participant's ability to perform basic tasks and understand health care information ("How confident are you filling out medical forms on your own?"). Another item from the Single Item Literacy Screener (33) was added to the health literacy scale to identify patients from diverse populations with limited reading ability (α = 0.80).

#### Perceived Barriers to Care

We asked participants about 11 barriers to behavioral health treatment at baseline (coded as 0 if not mentioned, and 1 if yes for each barrier) according to their previous experiences. If the participant reported not having any previous experience with accessing behavioral health services, the research assistant asked her/him to think about what barriers she/he thinks they would have encountered. The list of reported barriers was a combination of attitudinal and structural factors that may influence health care utilization according to the model. Attitudinal barriers (related to the individual's beliefs and values) included “wanting to handle a problem on one's own,” “thinking treatment would not work,” “concerns about stigmatization,” and “concerns about poor treatment because of one's ethnic/racial background.” Structural barriers (related to individual's perception of the health care system) included “fear of not knowing how to communicate problems because of language barriers,” “previous negative experiences with treatment,” “treatment cost,” and “problems with transportation and scheduling times.” Participants indicated whether they had experienced each barrier and had the opportunity to add additional barriers not described in the assessment.

#### Illness Level/Clinical Factors

Depression was measured with the Patient Health Questionnaire (PHQ-9) (α = 0.85) (34, 35), as assessed by DSM-IV diagnostic criteria of major depressive disorder. We also administered the brief General Anxiety Disorder scale (GAD-7) (α = 0.86) (36, 37), a seven-item clinical screener for generalized anxiety. To assess trauma, we included the Brief Trauma Questionnaire (BTQ) (38), a 10-item self-reported measure that examines experiences of potentially traumatic events, and the Post-Traumatic Stress Disorder Checklist (PCL-5) (α = 0.94) (39, 40), a self-reported measure of the DSM-V symptoms of PTSD. We also used the Hopkins Symptoms Checklist (HSCL-20) (α = 0.92) (41), a 20-item measure assessing symptoms of anxiety and depression. The resulting score is the average rating across all items (range: 0–4) with higher scores indicating more severe symptoms. Substance use was measured using: the Alcohol Use Disorders Identification Test (AUDIT) (α = 0.78) (42, 43), a World Health Organization screener for excessive drinking; the Drug Abuse Screening Test (DAST) (α = 0.87) (44, 45), a 10-item, yes/no self-reported instrument designed to screen for substance use; drug (α = 0.84, range: 0–1, cut-off: 0.1+) and alcohol sections (α = 0.70, range: 0–1, cut-off: 0.1+) of the Addiction Severity Index (ASI)-Lite (46) which evaluates lifetime and past 30-day behaviors; and a selection of eight items from the Benzodiazepine Dependence Questionnaire (BDEPQ) (α = 0.90) (47) that measure dependence to benzodiazepine tranquilizers, sedatives, and hypnotics. We measured smoking using the Fagerström Test for Nicotine Dependence (48, 49) (α = 0.75), a standard six-item instrument assessing the intensity of physical addiction to nicotine.

### Statistical Analysis

Our sample includes only participants from the intervention arm of the study (N = 172), divided into subgroups according to the number of treatment sessions they received: 0–1 sessions (tercile 1) (N = 48), 2–9 sessions (tercile 2) (N = 60), and 10–12 sessions (tercile 3) (N = 64). We assessed differences between the three subgroups, comparing proportions and means (with standard deviations) of sociodemographic, clinical measures, and sociocultural factors, by using bivariate regression and Pearson Chi-square tests for inter-group differences for continuous and categorical baseline characteristics. We also summarized individual barriers to care reported by participants and assessed subgroup differences using Chi-square tests with pairwise comparison of 2–9 treatment sessions (tercile 2) and 10–12 treatment sessions (tercile 3) *versus* those who attended 0–1 session (tercile 1 as reference category). Using multinomial logit models, we evaluated the associations between barriers to care and treatment adherence while controlling for demographic (age, gender, site, employment status), psychosocial (discrimination), and clinical (HSCL, benzodiazepine use scale, ASI alcohol scores at baseline) factors. To mitigate the issue of multi-collinearity among the baseline characteristics, our final model included those with significant subgroup differences at the 95% confidence level—site, benzodiazepine dependence, ASI alcohol, HSCL, and two measures of treatment barriers—as well as basic demographics (age, gender), employment status, and a measure of everyday discrimination, as we hypothesized that these would be significant predictors of immigrants' adherence to care (50). To address missing data in the HSCL instrument (19% of observations were missing because the instrument was administrated later in the study), we implemented multiple imputation methods using the "mi" procedure in Stata (51). This technique creates twenty complete datasets, imputes missing values using a chained equations approach, analyses each dataset, and uses standard rules (52) to combine estimates and adjust standard errors for uncertainty due to imputation. We also conducted a sensitivity analysis excluding the HSCL instrument from the multinomial regression. For missing values other than HSCL measure, we used list-wise deletion for (N = 3; 1.7%); so, the analytic sample for the multivariate analysis comprised 169 observations.

## Results

### Adherence to Intervention

**Table 1** summarizes the baseline sociodemographic characteristics of the sample and the three categories of treatment adherence. Across the three sites, 27.9% (N = 48) of participants attended 0–1 sessions, 34.9% attended (N = 60) 2–9 sessions, and 37.2% (N = 64) of participants completed the whole program, attending 10 or more sessions. Adherence across the recruitment sites was significantly different (*p* = 0.012), with Barcelona exhibiting the lowest rates. No significant differences in treatment adherence were found by age, gender, race/ethnicity, region of origin, socioeconomic status, employment, or education.

**Table 1:** Sociodemographic individual characteristics and adherence to intervention (N = 172).

	Total intervention sample (n = 172)		Treatment adherence tercile 1 (0–1 sessions) (n = 48)	Treatment adherence tercile 2 (2–9 sessions) (n = 60)	Treatment adherence tercile 3 (10–12 sessions) (n = 64)	Difference-test
	N	%	%	%	%	P-value
**Site**						
Boston	44	25.6	18.8	28.3	28.1	0.012
Madrid	41	23.8	10.4	25.0	32.8	
Barcelona	87	50.6	10.8	46.7	39.1	
**Age category**						
18–34	100	58.1	64.6	60.0	51.6	0.064
35–49	48	27.9	31.3	28.3	25.0	
50+	24	14.0	4.2	11.7	23.4	
**Gender**						
Male	78	45.3	52.1	43.3	42.2	0.539
Female	94	54.7	47.9	56.7	57.8	
**Race^†^**						
White	29	17.0	18.8	11.7	20.6	0.641
Black	9	5.3	4.2	8.3	3.2	
Indigenous/Native American	9	5.3	4.2	3.3	7.9	
Hispanic/Latino/Caribbean	21	12.3	8.3	15.0	12.7	
Mixed	103	60.2	64.6	61.7	55.6	
**Region of origin**						
United States/Spain	9	5.2	8.3	5.0	3.1	0.222
Central America and Mexico	27	15.7	8.3	21.7	15.6	
South America	115	66.9	77.1	61.7	64.1	
Caribbean	21	12.2	6.3	11.7	17.2	
**Education level**						
Less than high school	68	39.5	45.8	46.7	28.1	0.062
HS diploma, GED, vocational school, or more	104	60.5	54.2	53.3	71.9	
**Total personal income before tax in past year^††^**						
< US$15,000	142	83.5	91.3	83.3	78.1	0.184
≥US$15,000	28	16.5	8.7	16.7	21.9	
**Employment status**						
Unemployed	80	46.5	45.8	51.7	42.2	0.568
Employed	92	53.5	54.2	48.3	57.8	

^†^Column percentage summed across racial groups exceeds 100% due to rounding up to first decimal point.

^††^Reported total personal income before tax is not adjusted for cost of living in the two countries.

Participants randomized to receive the IIDEA intervention differed in terms of sociodemographic and clinical characteristics between the participants across the three sites (Boston, Madrid, and Barcelona) as well as between the participants in Spain (Madrid+Barcelona) and the US (Boston). **Table 1s** in the **Supplementary Material** reflects these differences. Overall, participants recruited in Spain were younger, with higher education level, mostly coming from South America (89.1%), being first generation immigrants without citizenship and lower sense of belonging while participants recruited in U.S. were older and reported being of Caribbean or Central American origin (including Mexico) in a much higher proportion (40.9% and 36.4%, respectively). Participants in the U.S. reported longer time from migration compared to those recruited in Spain and reported higher scores in the measures of discrimination, acculturation stress, depression, anxiety, trauma, and smoking but lower scores in the alcohol abuse (AUDIT) measure.

**Table 2** presents bivariate relationships between psychosocial, cultural and clinical factors, reported barriers to care, and treatment adherence. Adherence to the intervention was not related to psychosocial or cultural factors. Those who reported at least one barrier (89.0% of the sample) had significantly higher treatment adherence compared to those who reported no barriers (*p* = 0.007). Moreover, those who reported three or more barriers (59.3% of the sample) showed higher treatment adherence compared to those who reported zero, one, or two barriers (*p* = 0.004). Bivariate analyses of baseline clinical profile with treatment adherence groups (**Table 2**) showed that participants with higher scores on the HSCL-25 exhibited higher adherence to the program than those with lower scores (*p* = 0.027). The same pattern was observed for participants with higher scores on the BDEPQ (*p* = 0.009) and ASI Alcohol (*p* = 0.042). No significant associations were found regarding the other clinical factors (e.g., PHQ-9, PCL-5, Fagerström Test).

**Table 2:** Baseline barriers, social, cultural, and clinical factors and adherence to IIDEA (N = 172).

		Total IIDEA Sample (n = 172)	IIDEA adherence tercile 1 (0–1 sessions) (n = 48)	IIDEA adherence tercile 2 (2–9 sessions) (n = 60)	IIDEA adherence tercile 3 (10–12 sessions) (n = 64)	Difference-test
	n	Mean/%	Mean/%	Mean/%	Mean/%	P-value
**Generations**						
1st Generation	154	90.6%	87.2%	93.2%	90.6%	0.577
2nd Generation	16	9.4%	12.8%	6.8%	9.4%	
**Citizenship^†^**						
Noncitizen	78	46.2%	45.8%	49.2%	43.6%	0.825
Citizen	91	53.9%	54.2%	50.9%	56.5%	
**Sense of belonging^†^**						
No	70	40.9%	37.5%	40.7%	43.8%	0.800
Yes	101	59.1%	62.5%	59.3%	56.3%	
**Speak language in the host Country^†^**						
No	43	25.0%	18.8%	30.0%	25.0%	0.407
Yes	129	75.0%	81.3%	70.0%	75.0%	
**Health Literacy Scale**	170	12.51	12.54	12.36	12.63	0.863
Median		13.50	13.00	13.00	14.00	
Interquartile range		4.00	3.00	4.00	4.00	
**Discrimination Scale**	171	18.02	16.67	17.87	19.17	0.284
Median		16.00	14.00	16.00	17.50	
Interquartile range		9.00	9.50	8.00	11.88	
**Ethnic Identity Scale**	171	9.44	9.42	9.47	9.44	0.987
Median		10.00	10.00	10.00	9.00	
Interquartile range		3.00	2.00	3.00	3.00	
**Family Conflict Scale**	171	2.25	1.77	2.49	2.38	0.149
Median		2.00	1.00	2.00	2.00	
Interquartile range		3.00	3.00	3.00	3.00	
**Acculturative Stress Scale**	171	3.30	2.96	2.95	3.89	0.093
Median		3.00	2.00	2.00	4.00	
Interquartile range		4.00	3.50	3.00	3.50	
**Years in US/Spain**	154	10.03	9.40	10.65	9.90	0.776
**Reported barriers**						
Number of reported barriers <3	70	40.7%	60.4%	35.0%	31.3%	0.004
≥3 barriers reported	102	59.3%	39.6%	65.0%	68.8%	
**Any reported barriers**						
No	19	11.0%	22.9%	5.0%	7.8%	0.007
Yes	153	89.0%	77.1%	95.0%	92.2%	
**Clinical factors**						
Depression (PHQ-9)	172	10.88	9.98	10.85	11.58	0.318
Generalized anxiety (GAD-7)	172	8.53	7.19	8.97	9.13	0.081
PTSD (PCL)	172	27.19	22.88	28.87	28.86	0.113
Drug abuse (DAST)	170	1.27	1.61	1.33	0.97	0.294
Alcohol abuse (AUDIT)	172	5.20	5.52	5.33	4.84	0.577
Benzodiazepines (BDEPQ)	171	2.13	1.63	1.17	3.41	0.009
ASI Alcohol	172	0.22	0.16	0.22	0.26	0.042
ASI Drug	172	0.04	0.04	0.05	0.03	0.404
Hopkins Symptom Checklist (HSCL)^‡^	140	1.55	1.37	1.46	1.78	0.027
Smoking (Fagerström)	172	0.69	0.65	1.02	0.41	0.132
Reported trauma exposure						
No	8	4.7%	8.3%	0.0%	6.3%	0.092
Yes	164	95.3%	91.7%	100.0%	93.8%	

^†^Column percentage sum exceeds 100% due to rounding up to first decimal point.

^‡^The Hopkins Symptom Checklist was missing for 32 cases since the instrument was administrated later in the study.

### Reported Barriers and Adherence to the IIDEA Intervention

The association of perceived barriers to care and adherence to treatment is depicted in **Table 3**. Reported barriers of mistrust in previous treatment(s) (*p* = 0.007) and transportation or scheduling problems (*p* = 0.003) were both significant predictors of greater treatment adherence (10+ sessions *vs.* 0–1 sessions).

**Table 3:** Reported barriers to care and adherence to treatment (N = 172).

Reported barrier to care:		Total intervention sample (n = 172)	Treatment adherence tercile 1 (0–1 sessions) (n = 48)	Treatment adherence tercile 2 (2–9 sessions) (n = 60)	Treatment adherence tercile 3 (10–12 sessions) (n = 64)	Chi^2^ test (tercile 2 *vs.* tercile 1)	Chi^2^ test (tercile 3 *vs.* tercile 1)
	n	%	%	%	%	P-value	P-value
**Want to handle the problem on your own**							
No	56	32.6	39.6	23.3	35.9	0.077	0.693
Yes	115	66.9	60.4	75.0	64.1		
**Think the previous treatment would not work**							
No	90	52.3	66.7	51.7	42.2	0.105	0.007
Yes	80	46.5	31.3	46.7	57.8		
**Received treatment before and it didn't work**							
No	136	79.1	81.3	78.3	78.1	0.837	0.685
Yes	35	20.3	18.8	20.0	21.9		
**Concerned about how much money it would cost**							
No	104	60.5	68.8	56.7	57.8	0.237	0.279
Yes	66	38.4	31.3	41.7	40.6		
**Concerned about what people would think if they found out you were in treatment**							
No	98	57.0	64.6	53.3	54.7	0.279	0.292
Yes	73	42.4	35.4	45.0	45.3		
**Have problems with things like transportation or scheduling that made it hard to get to treatment**							
No	126	73.3	87.5	73.3	62.5	0.094	0.003
Yes	45	26.2	12.5	25.0	37.5		
**Were you unsure about where to go or who to see**							
No	93	54.1	64.6	53.3	46.9	0.279	0.063
Yes	78	45.3	35.4	45.0	53.1		
**Were you scared of being put in a hospital against your will**							
No	122	70.9	79.2	68.3	67.2	0.257	0.161
Yes	49	28.5	20.8	30.0	32.8		
**Were you concerned that you would be treated unfairly because of your race/ethnicity**							
No	139	80.8	79.2	81.7	81.3	0.608	0.784
Yes	32	18.6	20.8	16.7	18.8		
**Did you think you might not be able to communicate because of linguistic barrier**							
No	151	87.8	85.4	91.7	85.9	0.186	0.938
Yes	20	11.6	14.6	6.7	14.1		
**Is there any other obstacle you encountered^†^**							
No	166	96.5	97.9	95.0	96.9	0.684	0.735
Yes	5	2.9	2.1	3.3	3.1		

All barrier indicators are binary variables. Row frequencies and percent for positive outcome are reported.

^†^Other obstacles included severity of symptoms, undocumented status, and fear of discrimination due to sexual orientation.

Results of the association between barriers to care and treatment adherence after adjusting for psychosocial, demographic, and clinical factors are presented in **Table 4**. Participants who reported mistrust in previous treatment(s) had significantly higher odds of completing 10–12 treatment sessions (tercile 3) *vs.* 0–1 sessions (tercile 1) (OR = 3.79, CI = [1.49,9.66]). Transportation or scheduling problems, benzodiazepine dependence, discrimination, HSCL-25, and ASI Alcohol measures were not significantly related to treatment adherence in the multinomial logistic models after adjusting for other factors. A sensitivity analysis excluding the imputed HSCL-25 instrument yielded no substantial differences in the associations between the measures of barriers to care and treatment adherence.

**Table 4:** Odds ratios and 95% confidence intervals from a multinomial logistic regression model for the association between barriers to care and adherence to treatment (reference: treatment adherence tercile 1, or 0–1 sessions) (N = 169).

Characteristic	Treatment adherence tercile 2 (2–9 sessions)	Treatment adherence tercile 3 (10–12 sessions)
Think the previous treatment wouldn't work	2.23	3.79**
	[0.92,5.40]	[1.49,9.66]
Have problems with things like transportation or scheduling that made it hard to get to treatment	1.78	2.51
	[0.56,5.65]	[0.79,7.93]
Age group (ref: 18–34)	1	1
	[.,.]	[.,.]
35–49	0.86	0.47
	[0.32,2.31]	[0.16,1.44]
50+	2.26	4.16
	[0.36,14.07]	[0.68,25.35]
Female	1.29	1.02
	[0.52,3.21]	[0.39,2.67]
Site (re: Boston)	1	1
	[.,.]	[.,.]
Madrid	2.75	3.09
	[0.64,11.72]	[0.70,13.67]
Barcelona	0.63	0.57
	[0.20,2.03]	[0.17,1.92]
Employment status (ref: unemployed)	1	1
	[.,.]	[.,.]
Employed	0.7	0.98
	[0.30,1.65]	[0.40,2.42]
Benzodiazepine	0.91	1.01
	[0.79,1.04]	[0.89,1.15]
Discrimination	1	0.99
	[0.94,1.06]	[0.94,1.05]
ASI Alcohol	1.46	1.72
	[0.83,2.57]	[0.98,3.00]
Hopkins Symptom Checklist (HSCL)	1.23	1.58
	[0.64,2.37]	[0.79,3.13]
Sample size for treatment adherence group	58	64
Sample size for model	169	169

**P<0.01 (two-tailed tests). There are three cases that have at least one missing value and therefore are listwise deleted. The variable "ASI Alcohol" is standardized to mean = 0 and standard deviation = 1. Multiple imputation technique with chained equations was used to impute missing values for Hopkins Symptom Checklist (HSCL)—32 missing values were imputed.

## Discussion

To our knowledge, this is one of the few studies to prospectively examine perceived barriers to care associated with adherence to culturally-adapted cognitive behavioral interventions aimed at treating co-occurring mental health and substance use disorders among Latino immigrant populations. We did not find differences in treatment adherence related to employment, gender, or educational status across groups.

Symptom severity alone did not predict adherence after accounting for barriers to care. Although we found a significant positive association between greater severity of alcohol misuse and mood symptoms and treatment completion in the bivariate analysis, once we adjusted for the reported barriers and sociodemographic characteristics, the association was no longer statistically significant. So contrary to our hypothesis, clinical symptom severity did not significantly predict treatment adherence after accounting for barriers to care, as reported in other studies (6, 8). This finding may be due to differences in our sample composition. The previous studies finding severity of symptoms as a predictor of adherence included people with no or low levels of symptomatology while this trial required participants in the sample to have elevated levels of psychiatric symptomatology. For this group with already elevated symptomatology and involving co-morbidity, greater severity as reported by the respondent might not independently correlate with adherence to treatment. Another potential explanation is that fears of disclosing severe symptoms might lead to inaccurate reporting, thus making severity a noisy indicator of need for care. Fear of disclosing severe mental health symptoms is a common phenomenon among immigrant populations because of perceived risks, such as deportation and loss of employment (53, 54).

### Mistrust in Prior Treatment Uniquely Predicted Adherence

Contrary to our expectations, individuals who reported more barriers had higher rates of adherence to treatment than those who reported fewer barriers. Of all the barriers assessed, mistrust in prior treatment most strongly predicted adherence. Individuals who reported “thinking that the previous treatment would not work” showed higher rates of completion than those not reporting this barrier, after adjusting for clinical factors (including severity), discrimination, and demographic characteristics. This surprising finding may be due the intervention's accessibility and culturally specific design.

The IIDEA program used several strategies to address access and cultural needs of Latino immigrants, including providing care in community-based organizations, allowing greater flexibility in scheduling sessions (including nights and weekends, providing sessions by phone or in community settings), administering treatment in patients' preferred language, and using extensive follow-up procedures (e.g., calling before sessions). These features, uncommon in standard treatment options, might have appealed to individuals who do recognize a need for care, but previously found traditional services to be inaccessible, inflexible, and culturally incompetent.

Cultural matching between patients and providers is key in addressing the barrier of cultural mistrust in treatment, and the integration of these culturally competent approaches in the IIDEA intervention may explain why participants with this barrier continued treatment compared to those without mistrust. Previous research has shown that linguistic matching impacts therapeutic alliance and improves quality of care (55). Culturally tailored interventions have shown to improve treatment engagement and adherence among Latino populations (56, 57), particularly when the adaptation utilizes metaphors and symbols matching the patient's worldview, considers symptom attributions, and implicitly addresses cultural factors (58). A meta-analysis of cultural matching between therapists and patients (59) found that patients showed a strong preference for therapists of their own ethnicity and a tendency to perceive them more positively. There is also the possibility that participants who did not report mistrust in prior services might be dropping the intervention at early stages for other reasons, such as lack of time and competing demands of childcare (60).

### Strengths and Weaknesses of the Study

The present study has several strengths. It highlights the importance of exploring barriers to care before initiating therapeutic interventions with Latino immigrants. Findings contribute to the evidence on addressing cultural mistrust and improving treatment adherence using intensive but flexible community-based approaches with culturally-tailored interventions. An additional strength of the study was the wide heterogeneity of Latinos (i.e., 17 different countries of origin) across the recruitment sites.

Our study has several limitations. First, we did not assess problem recognition. Some individuals with a high number of barriers to care who did not initiate treatment may be unaware of having a mental health or substance use problem and we would be unable to capture them in our study. These individuals could drop out of treatment early, unaware of the potential benefits of treatment. We acknowledge that the sample used in the analysis is relatively small and future studies should attempt to replicate the analysis with larger samples. We also recognize that cultural matching between provider and participant is often not possible in community-based settings. We recommend that future studies evaluate whether ethnic and linguistic mismatch by providers and participants impact adherence to the intervention. Despite these limitations, the study offers important insight on the importance of exploring barriers to treatment and tailoring interventions to specific ethnic groups.

## Conclusions

This study provides valuable information on how mistrust in behavioral health services might influence treatment adherence in an evidence-based, culturally-centered intervention for Latino immigrants with co-occurring disorders. The present findings support ethnic and linguistic matching between patients and providers as effective methods for tackling attitudinal barriers to behavioral health treatment. Specifically, these methods could assist Latino immigrants in overcoming cultural mistrust toward services and increase their adherence to treatment. Our findings also suggest that exploring barriers to treatment prior to initiating treatment may allow clinicians to better address individual perceptions that treatment might not work, and consequently could impact improved adherence.

## Data Availability Statement

The datasets for this manuscript are not publicly available because it involves an ethnic minority sample including participants with substance use. We are not able to release data as part of the publication, given the sensitivity of the data, and our agreement with the institutions' IRB.

Requests to access the datasets should be directed to Sheri Markle, smarkle@mgh.harvard.edu.

## Ethics Statement

The studies involving human participants were reviewed and approved by Institutional Review Board, Massachusetts General Hospital, Boston, Massachusetts, USA Institutional Review Board, University Hospital Parc Tauli-I3PT, Sabadell, Spain Institutional Review Board, Universitat Autonoma de Barcelona, Barcelona, Spain Institutional Review Board, CIBERSAM, Barcelona, Spain. The patients/participants provided their written informed consent to participate in this study.

## Author Contributions

MA, DV, and IF-B contributed to the conception and design of the study. YW and SB organized the database and performed the statistical analysis. IF-B and MA wrote the first draft of the manuscript. NA and KD wrote sections of the manuscript. All authors contributed to manuscript revision and approved the submitted version.

## Funding

International Latino Research Partnership. Research reported in this publication was supported by the National Institute on Drug Abuse (NIDA) of the National Institutes of Health under Award Number R01DA034952. The content is solely the responsibility of the authors and does not necessarily represent the official views of the National Institutes of Health.

## Conflict of Interest

The authors declare that the research was conducted in the absence of any commercial or financial relationships that could be construed as a potential conflict of interest.

## Supplementary Material

The rest of the supplementary material can be found at this Figshare link: https://doi.org/10.6084/m9.figshare.10087292.v2
