# Supplementary material for: Predictors of Adherence to Treatment in Behavioral Health Therapy for Latino Immigrants: The Importance of Trust
Source: Front Psychiatry. 2019 Nov 8;10:817. doi: 10.3389/fpsyt.2019.00817 (PMC6856783; doi:10.3389/fpsyt.2019.00817)
Supplement: Supplementary file 1 [file DataSheet_1.docx]

***Supplementary Materials***

**Figure 1s.** Distribution of adherence to treatment

| **Table 1s:** Socio-demographics, Clinical and Cultural Characteristics Among Intervention Patients by site (N=172) | | | | | | | | | |
| --- | --- | --- | --- | --- | --- | --- | --- | --- | --- |
|  |  | **All site (n=172)** | **Boston  (n=44)** | **Madrid (n=41)** | **Barcelona (n=87)** | **Variance across 3 sites** | **Spain**  **[Madrid+**  **Barcelona] (n=128)** | **US**  **[Boston]**  **(n=44)** | **Variance Spain v. US** |
|  | N | mean or % | mean or  % | mean or  % | mean or  % | P-value | mean or  % | mean or  % | P-value |
| Treatment adherence |  |  |  |  |  |  |  |  |  |
| Tertile 1  (0-1 session) | 48 | 27.9% | 20.5% | 12.2% | 39.1% | 0.012 | 30.5% | 20.5% | 0.442 |
| Tertile 2  (2-9 sessions) | 60 | 34.9% | 38.6% | 36.6% | 32.2% |  | 33.6% | 38.6% |  |
| Tertile 3  (10-12 sessions) | 64 | 37.2% | 40.9% | 51.2% | 28.7% |  | 35.9% | 40.9% |  |
| **Sociodemographic Characteristics** |  |  |  |  |  |  |  |  |  |
| Age^†^ |  |  |  |  |  |  |  |  |  |
| 18-34 | 100 | 58.1% | 40.9% | 46.3% | 72.4% | 0.001 | 64.1% | 40.9% | 0.011 |
| 35-49 | 48 | 27.9% | 34.1% | 31.7% | 23.0% |  | 25.8% | 34.1% |  |
| 50+ | 24 | 14.0% | 25.0% | 22.0% | 4.6% |  | 10.2% | 25.0% |  |
| Gender |  |  |  |  |  |  |  |  |  |
| Male | 78 | 45.3% | 36.4% | 36.6% | 54.0% | 0.069 | 48.4% | 36.4% | 0.165 |
| Female | 94 | 54.7% | 63.6% | 63.4% | 46.0% |  | 51.6% | 63.6% |  |
| Race^†^ |  |  |  |  |  |  |  |  |  |
| White | 29 | 17.0% | 23.3% | 12.2% | 16.1% | 0.000 | 14.8% | 23.3% | 0.000 |
| Black | 9 | 5.3% | 4.7% | 4.9% | 5.8% |  | 5.5% | 4.7% |  |
| Indigenous/native American | 9 | 5.3% | 4.7% | 7.3% | 4.6% |  | 5.5% | 4.7% |  |
| Hispanic/Latino/  Caribbean | 21 | 12.3% | 46.5% | 2.4% | 0.0% |  | 0.8% | 46.5% |  |
| Mixed | 103 | 60.2% | 20.9% | 73.2% | 73.6% |  | 73.4% | 20.9% |  |
| Region of Origin^†^ |  |  |  |  |  |  |  |  |  |
| United States/Spain | 9 | 5.2% | 20.5% | 0.0% | 0.0% | 0.000 | 0.0% | 20.5% | 0.000 |
| Central America | 27 | 15.7% | 36.4% | 7.3% | 9.2% |  | 8.6% | 36.4% |  |
| South America | 115 | 66.9% | 2.3% | 87.8% | 89.7% |  | 89.1% | 2.3% |  |
| Caribbean | 21 | 12.2% | 40.9% | 4.9% | 1.1% |  | 2.3% | 40.9% |  |
| Education level |  |  |  |  |  |  |  |  |  |
| Less than High School | 68 | 39.5% | 52.3% | 39.0% | 33.3% | 0.111 | 35.2% | 52.3% | 0.045 |
| HS Diploma, GED, Vocational School, or More | 104 | 60.5% | 47.7% | 61.0% | 66.7% |  | 64.8% | 47.7% |  |
| Total personal income before tax^††^ |  |  |  |  |  |  |  |  |  |
| < US $15,000 | 142 | 83.5% | 59.1% | 95.1% | 90.6% | 0.000 | 92.1% | 59.1% | 0.000 |
| ≥ US $15,000 | 28 | 16.5% | 40.9% | 4.9% | 9.4% |  | 7.9% | 40.9% |  |
| Employment Status |  |  |  |  |  |  |  |  |  |
| Unemployed | 80 | 46.5% | 47.7% | 39.0% | 49.4% | 0.536 | 46.1% | 47.7% | 0.851 |
| Employed | 92 | 53.5% | 52.3% | 61.0% | 50.6% |  | 53.9% | 52.3% |  |
| Generations^†^ |  |  |  |  |  |  |  |  |  |
| 1st Generation | 154 | 90.6% | 69.1% | 95.1% | 98.9% | 0.000 | 97.7% | 69.1% | 0.000 |
| 2nd Generation | 16 | 9.4% | 31.0% | 4.9% | 1.2% |  | 2.3% | 31.0% |  |
| **Clinical Profile at Baseline** |  |  |  |  |  |  |  |  |  |
| Depression (PHQ-9) | 172 | 10.88 | 12.41 | 12.22 | 9.47 | 0.003 | 10.35 | 12.41 | 0.033 |
| Generalized Anxiety (GAD-7) | 172 | 8.53 | 10.00 | 9.34 | 7.40 | 0.007 | 8.02 | 10.00 | 0.021 |
| PTSD (PCL) | 172 | 27.19 | 31.41 | 30.46 | 23.52 | 0.014 | 25.74 | 31.41 | 0.054 |
| Drug Abuse (DAST) | 170 | 1.27 | 1.59 | 0.95 | 1.26 | 0.390 | 1.16 | 1.59 | 0.251 |
| Alcohol Abuse (AUDIT) | 172 | 5.20 | 4.18 | 4.93 | 5.85 | 0.034 | 5.55 | 4.18 | 0.027 |
| Benzodiazepines (BDEPQ) | 171 | 2.13 | 1.86 | 3.54 | 1.61 | 0.052 | 2.23 | 1.86 | 0.629 |
| ASI Alcohol | 172 | 0.22 | 0.21 | 0.24 | 0.22 | 0.802 | 0.22 | 0.21 | 0.664 |
| ASI Drug | 172 | 0.04 | 0.06 | 0.05 | 0.03 | 0.049 | 0.03 | 0.06 | 0.053 |
| Hopkins Symptom Checklist (HSCL) ^‡^ | 140 | 1.55 | 1.76 | 1.63 | 1.41 | 0.069 | 1.48 | 1.76 | 0.062 |
| Smoking (Fagerström) | 172 | 0.69 | 1.34 | 0.46 | 0.46 | 0.011 | 0.46 | 1.34 | 0.003 |
| Reported Trauma Exposure |  |  |  |  |  |  |  |  |  |
| No | 8 | 4.7% | 4.5% | 7.3% | 3.4% | 0.624 | 4.7% | 4.5% | 0.969 |
| Yes | 164 | 95.3% | 95.5% | 92.7% | 96.6% |  | 95.3% | 95.5% |  |
| **Cultural/Social Factors** |  |  |  |  |  |  |  |  |  |
| Citizenship^†^ |  |  |  |  |  |  |  |  |  |
| Noncitizen | 78 | 46.2% | 31.0% | 36.6% | 58.1% | 0.006 | 45.3% | 27.9% | 0.023 |
| Citizen | 91 | 53.9% | 69.1% | 63.4% | 41.9% |  | 54.7% | 72.1% |  |
| Sense of Belonging |  |  |  |  |  |  |  |  |  |
| No | 70 | 40.9% | 27.9% | 39.0% | 48.3% | 0.081 | 45.3% | 27.9% | 0.045 |
| Yes | 101 | 59.1% | 72.1% | 61.0% | 51.7% |  | 54.7% | 72.1% |  |
| Speak Language in the Host Country |  |  |  |  |  |  |  |  |  |
| No | 43 | 25.0% | 88.6% | 4.9% | 2.3% | 0.000 | 3.1% | 88.6% | 0.000 |
| Yes | 129 | 75.0% | 11.4% | 95.1% | 97.7% |  | 96.9% | 11.4% |  |
| Recruitment Site |  |  |  |  |  |  |  |  |  |
| Primary Care | 75 | 43.6% | 61.4% | 56.1% | 28.7% | 0.000 | 37.5% | 61.4% | 0.003 |
| Community Agency | 40 | 23.3% | 27.3% | 7.3% | 28.7% |  | 21.9% | 27.3% |  |
| Emergency Room | 10 | 5.8% | 0.0% | 24.4% | 0.0% |  | 7.8% | 0.0% |  |
| Referred | 47 | 27.3% | 11.4% | 12.2% | 42.5% |  | 32.8% | 11.4% |  |
| Health Literacy Scale | 170 | 12.51 | 11.24 | 12.34 | 13.21 | 0.001 | 12.93 | 11.24 | 0.001 |
| Discrimination Scale | 171 | 18.02 | 21.26 | 18.30 | 16.29 | 0.005 | 16.93 | 21.26 | 0.003 |
| Ethnic Identity Scale | 171 | 9.44 | 9.47 | 9.22 | 9.54 | 0.668 | 9.44 | 9.47 | 0.934 |
| Family Conflict Scale | 171 | 2.25 | 2.74 | 2.63 | 1.82 | 0.016 | 2.08 | 2.74 | 0.060 |
| Acculturative Stress Scale | 171 | 3.30 | 4.16 | 3.56 | 2.76 | 0.016 | 3.02 | 4.16 | 0.017 |
| Years in US/Spain | 154 | 10.03 | 17.10 | 12.53 | 6.45 | 0.000 | 8.31 | 17.10 | 0.000 |
| Number of Home Visits Past 12 Months | 171 | 0.20 | 0.19 | 0.20 | 0.21 | 0.969 | 0.20 | 0.19 | 0.832 |
| **Reported Barriers** |  |  |  |  |  |  |  |  |  |
| Number of reported barriers < 3 | 70 | 40.7% | 25.0% | 39.0% | 49.4% | 0.026 | 46.1% | 25.0% | 0.014 |
| >=3 barriers reported | 102 | 59.3% | 75.0% | 61.0% | 50.6% |  | 53.9% | 75.0% |  |
| **Other Reported Barriers** |  |  |  |  |  |  |  |  |  |
| No | 19 | 11.0% | 11.4% | 19.5% | 6.9% | 0.104 | 10.9% | 11.4% | 0.938 |
| Yes | 153 | 89.0% | 88.6% | 80.5% | 93.1% |  | 89.1% | 88.6% |  |

Notes:

^†^ Column percentage sum exceeds 100% due to rounding up to first decimal point.

^††^ Reported total personal income before tax is not adjusted for cost of living in the two countries.

^‡^ The Hopkins Symptom Checklist was missing for 32 cases since the instrument was administrated later in the study.
